# A comparison of radiographic techniques and electromagnetic transponders for localization of the prostate

**DOI:** 10.1186/1748-717X-7-101

**Published:** 2012-06-21

**Authors:** Ryan D Foster, David A Pistenmaa, Timothy D Solberg

**Affiliations:** 1 Department of Radiation Oncology, UT Southwestern Medical Center, 5801 Forest Park Road, Dallas, TX 75390-9183, USA

**Keywords:** Prostate, Localization, Electromagnetic transponders, Conebeam CT

## Abstract

**Background:**

The aim of this study is to compare three methodologies of prostate localization and to determine if there are significant differences in the techniques.

**Methods:**

Daily prostate localization using cone beam CT or orthogonal kV imaging has been performed at UT Southwestern Medical Center since 2006. Prostate patients are implanted with gold seeds, which are matched with the planning CT or DRR before treatment. More recently, a technology using electromagnetic transponders implanted within the prostate was introduced into our clinic (Calypso®). With each technology, patients are localized initially using skin marks and the room lasers. In this study, patients were localized with Calypso and either CBCT or kV orthogonal images in the same treatment session, allowing a direct comparison of the technologies. Localization difference distributions were determined from the difference in the offsets determined by CBCT/kV imaging and Calypso. CBCT-Calypso and kV imaging-Calypso localization data were summarized from over 900 and 250 fractions each, respectively. The Wilcoxon signed rank test is used to determine if the localization differences are statistically significant. We also calculated Pearson’s product–moment correlation coefficient (R^2^) to determine if there is a linear relationship between the shifts determined by Calypso and the radiographic techniques.

**Results:**

The differences between CBCT-Calypso and kV imaging-Calypso localizations are −0.18 ± 2.90 mm, -0.79 ± 2.18 mm, -0.01 ± 1.20 mm and −0.09 ± 1.40 mm, 0.48 ± 1.50 mm, 0.08 ± 1.04 mm, respectively, in the AP, SI, and RL directions. The Pearson product–moment correlation coefficients for the CBCT-Calypso shifts were 0.71, 0.92 and 0.88 and for the OBI-Calypso comparison were 0.95, 0.89 and 0.85. The percentage of localization differences that were less than 3 mm were 86.1%, 84.5% and 96.0% for the CBCT-Calypso comparison and 95.8%, 94.3% and 97% for the kV OBI-Calypso comparison. No trends were observed in the Bland-Altman analysis.

**Conclusions:**

Localization of the prostate using electromagnetic transponders agrees well with radiographic techniques and each technology is suitable for high precision radiotherapy. This study finds that there is more uncertainty in CBCT localization of the prostate than in 2D orthogonal imaging, but the difference is not clinically significant.

## Background

One of the greatest challenges in radiation oncology is the uncertainty of tumor and organ position within the patient. Computed tomography (CT) scans used for treatment planning are snapshots of the patient taken days before treatment begins. In the case of prostate cancer, variable filling of the bladder and rectum virtually guarantee that on the first day of treatment the prostate will not be in the same position as the day of the planning CT scan. To account for setup uncertainty and organ motion, the ICRU[1,2] has recommended that a margin be added to the target during the planning process. Unfortunately, the planning target volume (PTV) often includes healthy tissues or organs that are irradiated unnecessarily. If the prostate could be localized more accurately, the margin could be reduced and more healthy tissue could be spared. Accurate localization is even more critical for dose escalation and hypo-fractionation.

Prostate localization has been studied extensively and several different technologies have been used for daily localization, including transabdominal ultrasound, X-ray portal imaging, and kilovoltage and megavoltage cone beam CT [3-14]. With these technologies, patients are localized using bony anatomy, implanted fiducials, or three-dimensional volumetric images. These image-guided technologies have allowed the therapist to determine the magnitude and direction of the setup error and correct for it before treatment is initiated. Numerous studies have been published describing the merits of the various methods; in general, localization uncertainties are on the order of 0.5 cm (1σ) in each direction [6-14]. Prostate localization using implanted markers is increasing in use [7,9-11,14]. After conebeam CT (CBCT) or kV imaging, the markers are matched to a reference CT or a digitally reconstructed radiograph (DRR), and a software algorithm is used to calculate the necessary couch shifts.

The Calypso® 4D localization system (Varian Medical Systems, Palo Alto, CA) is the first localization technology to provide completely objective localization as well as continuous monitoring of the prostate position during radiation therapy. By utilizing electromagnetic detection of three transponders implanted in the prostate, Calypso provides a user- independent method of localizing the prostate gland in patients.

The purpose of this study is to compare the accuracy of CBCT, kV imaging and Calypso for localization of the prostate at a single institution.

## Methods and materials

Since 2006, prostate patients in our department have had gold seeds implanted for the purpose of localization, first with megavoltage imaging and more recently, kilovoltage imaging. Prostate patients are treated on a Varian Trilogy (Varian Medical Systems, Palo Alto, CA) or an Elekta SynergyS (Elekta Oncology Systems, Crawley, UK), with the decision based on whether or not the patient is receiving lymph node irradiation. Because our Elekta has the optional beam modulator with 4 mm wide leaves and a maximum field size of 21 x 16 cm^2^, all patients receiving lymph node irradiation are treated on the Varian.

Prostate patients undergo CT-simulation with a vacuum bag around the thighs and knees for immobilization, a rectal marker in place and a full bladder. CT slice thickness is 1.5 mm for patients with implanted Calypso Beacons. The prostate and proximal seminal vesicles are contoured and combined, constitute the CTV. A uniform PTV expansion of 6 mm is added to the CTV, except posteriorly, which has a 4 mm margin. The PTV receives 79.2 Gy in 44 fractions. If pelvic lymph nodes are treated, they receive 45 Gy in 25 fractions, after which, only the prostate and seminal vesicles receive treatment. Patients are asked to have a full bladder at the time of treatment, but are given no dietary instructions. However, if at the time of CT simulation, the patient was found to have a full or distended rectum, he was asked to void and was re-simulated.

Both linacs have kV on-board imaging (OBI) and are capable of performing conebeam CT (CBCT). Because of a disparate workload on the two machines, orthogonal kV imaging is the primary localization method on the Trilogy. On both machines, the patient is set up on the treatment couch by aligning the room lasers to skin marks. CBCT or kV images are then aligned manually to the implanted markers on the planning CT or the DRRs from the treatment planning system. The couch shifts are calculated based on the seed alignments and the patient’s position is adjusted accordingly. Our therapists have extensive experience using these technologies, as there have been over 3200 prostate localizations in our department since 2006.

The Calypso system uses an array of AC magnetic coils to generate a resonant response from implanted electromagnetic transponders (Beacons™), which is detected by a second array of receiver coils [15]. The array position relative to the linac isocenter is determined by three infrared cameras mounted on the ceiling in the room. Daily quality assurance is performed each morning and the entire system is calibrated monthly. The Beacons (8 mm in length and 2 mm in diameter) are implanted in the right and left base and the apex of the prostate. The implantation is performed with transrectal ultrasound guidance in a manner analogous to gold marker implantation. The coordinates of the Beacons are identified on the treatment planning CT by carefully contouring the Beacons and having the treatment planning software autoplace a point in the center of each contoured volume. The coordinates of these points and the isocenter are entered into the Calypso tracking station. Similar to CBCT and kV imaging, initial patient localization is performed using skin marks to align with room lasers. Calypso is used to localize the patient and the system calculates the initial offset. The couch is shifted until the three offsets are zero. During treatment, Calypso monitors and reports the offset between the actual and planned isocenter position at a rate of 10 Hz. Calypso has been shown to have sub-millimeter accuracy [15]. Additionally, Calypso localization compares favorably to that using room mounted kV x-ray localization of implanted radio-opaque markers [16], with a positional stability of the transponders that is similar to that of gold seeds [17].

Current CBCT-Calypso data consists of 915 treatment sessions from 21 patients and kV imaging-Calypso data consists of 260 localizations from 6 patients. For Calypso patients treated on the SynergyS, the CBCT is acquired and the couch shifts are calculated using the alignment of the Beacons to their contours from the planning system. Prior to moving the patient, Calypso is used to verify the CBCT shifts and the patient is then shifted to the correct position. Calypso patients treated on the Trilogy are localized using Calypso, shifted, and then kV images are taken. The alignment of the Calypso Beacon contours in the kV images is used to confirm the Calypso localization. In April 2010, we reduced the frequency of radiographic imaging of Calypso patients to once per week.

### Statistical analysis

Localization differences are calculated from the couch shifts determined by Calypso and the radiographic localization technique. The Wilcoxon signed rank test is used to determine if these differences are statistically significant and p values < 0.05 were considered statistically significant. Using the test proposed by Bland and Altman [18] for comparing two measurement methods, we plotted the difference in the couch shifts vs. the average of the shifts to try to discern any trends in the localization differences. If the methods are equivalent, the mean differences should be zero and there should be no trends observed in the data. We also calculated Pearson’s product–moment correlation coefficient (R^2^) to determine if there is a linear relationship between the shifts determined by Calypso and the radiographic techniques. Statistical analysis was performed using MatLab, version 7.8 (The MathWorks, Natick, MA).

## Results

**Figure 1:**
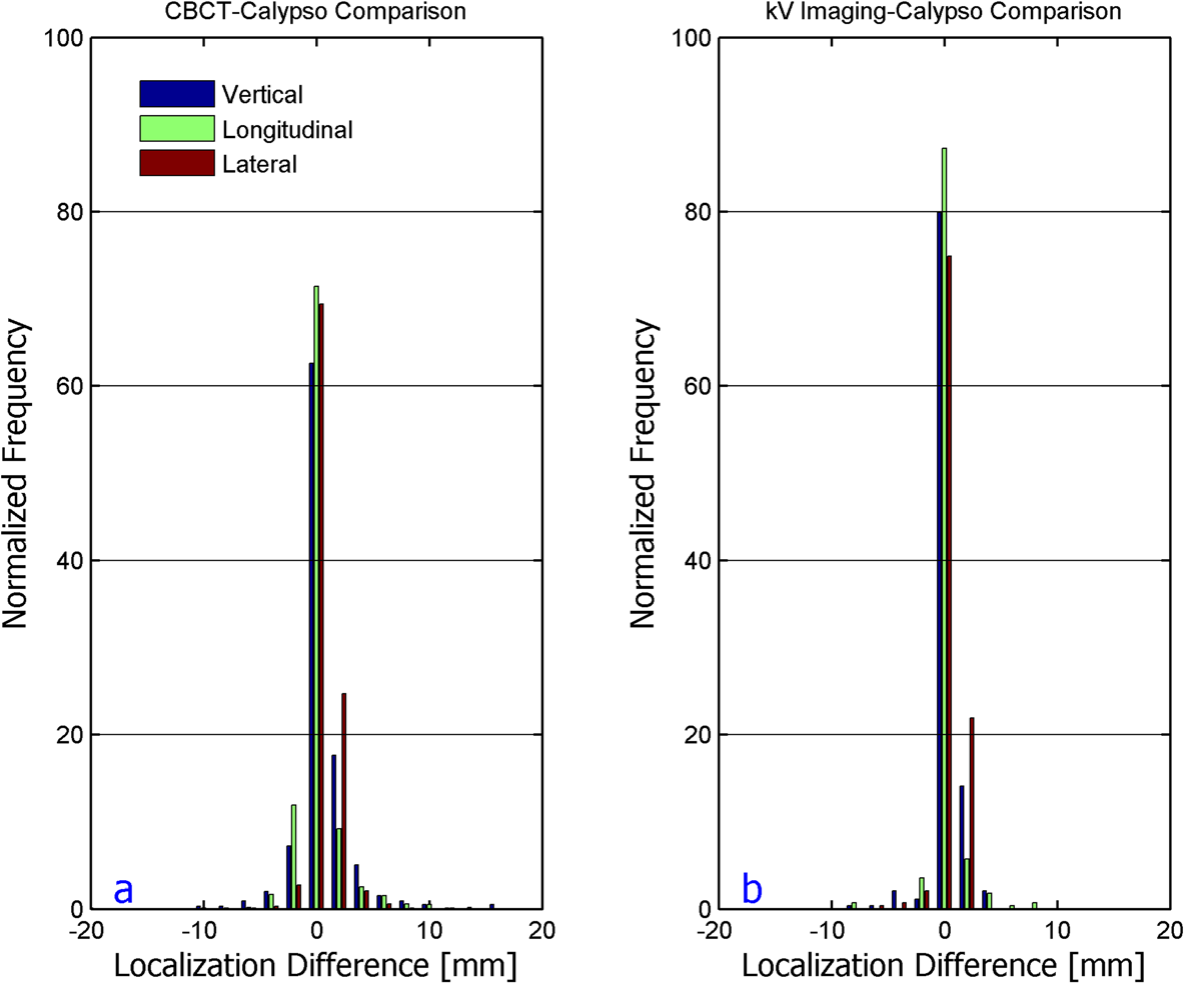
Localization difference distributions for CBCT - Calypso and kV-Imaging - Calypso comparison.

The average difference in the CBCT-Calypso and the kV imaging-Calypso localizations are shown in Table 1. The radiographic and Calypso localizations agree very well, with a difference of less than 1 mm in all directions. The distributions of the localization error differences are shown in Figure 1. For the combined population of patients, there were small but statistically significant differences (p < 0.05) in radiographic and Calypso localizations in the AP and SI directions, respectively. When the kV imaging and CBCT patients were analyzed separately, we again observed small, but statistically significant differences in the longitudinal and lateral directions for kV imaging and in the vertical and longitudinal directions for CBCT. Results of the Wilcoxon signed rank test are found in Table 2.

**Table 1:** Mean localization differences for OBI-Calypso and CBCT-Calypso (mm) comparisons and percentage of differences ≤ 3 mm

	**kV Imaging**		**CBCT**	
	Mean	SD	Mean	SD
Vert	−0.09 (95.8%)	1.40	−0.18 (86.1%)	2.90
Long	0.48 (94.3%)	1.50	−0.79 (84.5%)	2.18
Lat	0.08 (97.0%)	1.04	0.01 (96.0%)	1.20

**Table 2:** P values for the Wilcoxon signed rank test. Values less than 0.05 are considered significant

**Data Set**	**Vertical**	**Longitudinal**	**Lateral**
Combined	0.0046	< .0001	0.220
kV orthogonal imaging	0.865	< .0001	.001
CBCT	0.0028	< .0001	0.947

**Figure 2:**
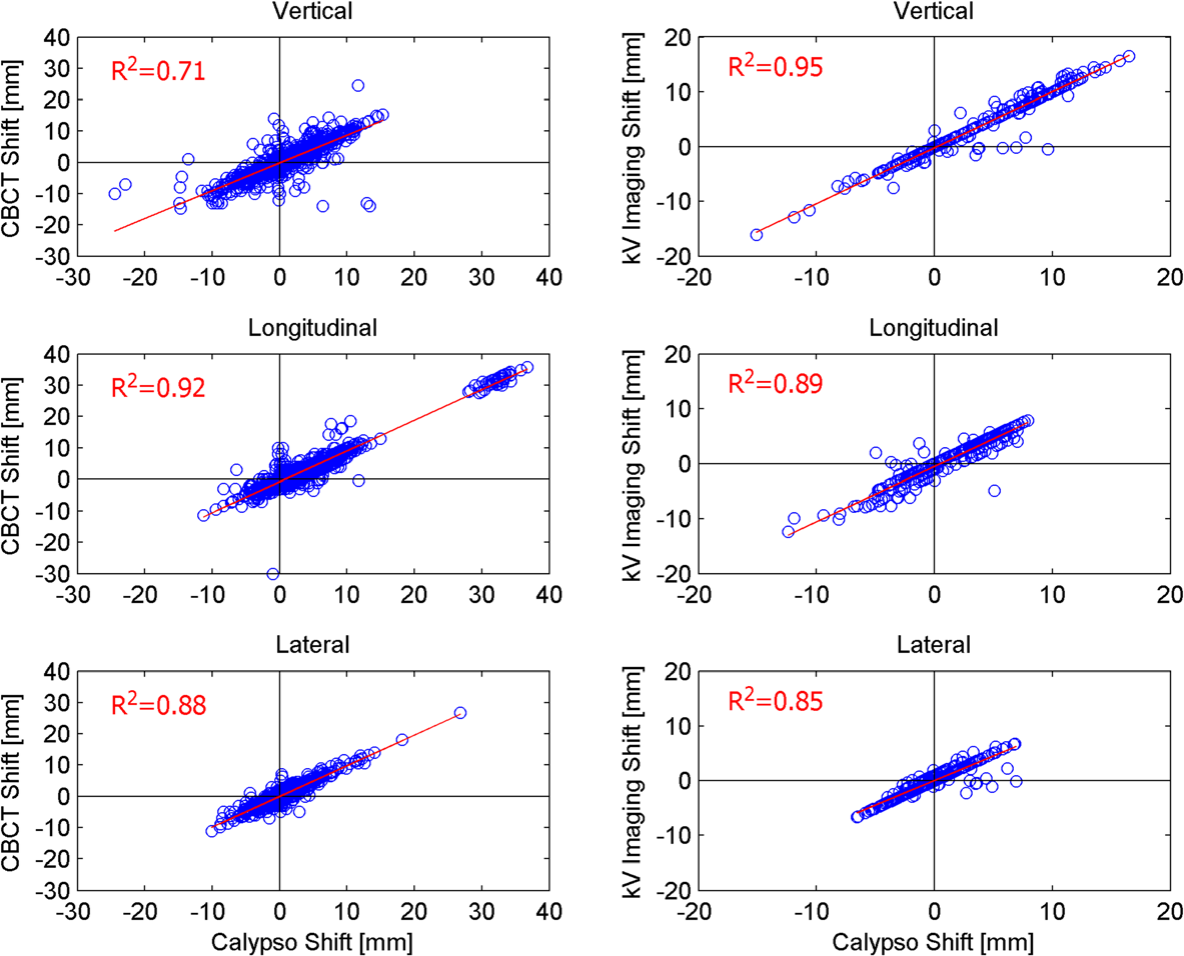
Plots of Calypso shifts vs radiographic shifts for the CBCT (left column) and kV imaging (right column) for the vertical, longitudinal and lateral directions.

**Figure 3:**
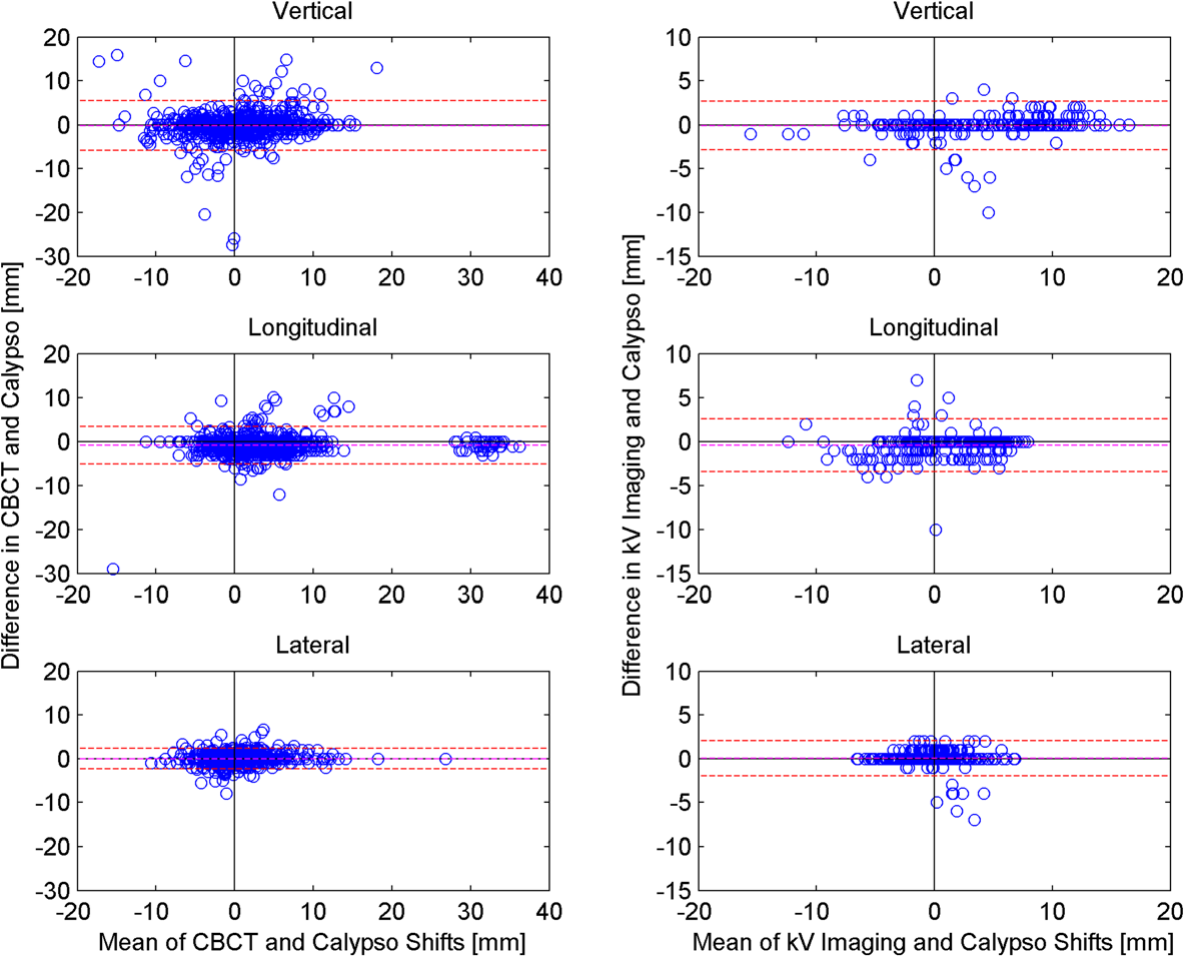
**Bland Altman plots of the difference in Calypso and radiographic shifts vs. the mean of the shifts for the Calypso-CBCT comparison (left column) and the Calypso-kV Imaging comparison (right column) for the vertical, longitudinal and lateral directions.** The red dashed lines represent the 95% limits of agreement and the magenta dashed line is the mean.

Figure 2 shows the couch shifts determined from the radiographic localizations plotted against those determined by Calypso. The plots for the kV imaging appear to lie on distinct lines due to the coarse resolution of the OBI shifts (0.1 cm). The Pearson correlation coefficients for the kV imaging data are 0.95, 0.89 and 0.85 in the vertical, longitudinal and lateral directions and for the CBCT data, are 0.71, 0.92 and 0.88, indicating a reasonably strong linear relationship between Calypso and the radiographic techniques. A correlation coefficient of 1 indicates a perfect linear dependence. The Bland-Altman plots for the vertical, lateral, and longitudinal couch shifts are shown in Figure 3. Also plotted are the mean difference (dashed magenta line) and the 95% limits of agreement (dashed red lines). The mean differences and 95% limits of agreement for the kV imaging were −0.81 (−2.83, 2.67), -0.41 (−3.41, 2.59) and 0.06 (−1.97, 2.09) mm for the vertical, longitudinal and lateral directions. For the CBCT, the mean differences were −0.18, -0.79 and −0.01 mm and the 95% limits of agreement were (−5.86, 1.01), (−5.06, 3.47) and (−2.37, 2.35) mm.

## Discussion

The use of implanted fiducial markers and conebeam CT has been shown to improve prostate localization and reduce interobserver differences when compared to bony anatomy and soft tissue alignment methods [19-21]. However, the question remains as to whether it is an improvement over 2D orthogonal radiographic imaging. This study is the first to investigate all three localization techniques and attempts to determine if there are significant differences among the three technologies.

Ogunleye et al. [22] studied Calypso and kV imaging differences for prostate localization and found mean differences of 1.2 ± 0.9, 1.1 ± 0.9 and 0.7 ± 0.5 mm in the vertical, longitudinal and lateral directions, respectively. Their results are slightly larger than other comparisons of Calypso and 2D radiographic imaging, which all found sub-millimeter differences between the technologies [16,23,24]. It is unclear what caused the somewhat larger differences in Ogunleye’s study despite their efforts to record Calypso and kV imaging positions at the same time.

A study analyzing Calypso-CBCT agreement found mean differences between those technologies that were larger than our results in the vertical and lateral directions [25]. They also found better average agreement between Calypso localization and CBCT alignment with bony anatomy in the vertical and lateral directions compared to CBCT alignment to the Beacons, which is unusual considering the unreliable nature of bony anatomy localization for the prostate [7,13]. However, their calculated theoretical PTV margins were larger for the bony anatomy alignment than for the other methods.

The distribution of the localization differences is wider for the CBCT-Calypso comparison than for the kV imaging-Calypso comparison and the mean localization differences are larger than those for kV imaging. These results suggest that there is more uncertainty in aligning fiducial markers using a 3D CBCT than in aligning them using two orthogonal images. One possible reason for this observation is that the Calypso localization was performed after CBCT imaging, but before kV OBI imaging, in the respective group of patients. It is possible that the prostate could move during CBCT reconstruction and analysis, resulting in a larger disagreement between Calypso and CBCT. Because the planar kV imaging process is faster than that of CBCT, there is a lower probability that the prostate will move between localizations. It is important to note that the therapists are not matching the fiducials directly on either the CBCT or planar kV images, but align the Beacon contours defined on the treatment planning CT with the Beacons in the kV and CBCT images. These same contours were used to determine the Beacon positions relative to the isocenter for the electromagnetic localization, therefore any systematic errors due to the definition of the Beacon positions should be present in all three techniques.

CBCT slice thickness also contributes to a greater uncertainty inherent in CBCT localization than in 2D fiducial marker localization. Owen et al. [26] found mean differences in fiducial marker localization between MV electronic portal imaging (EPI) and CT on rails to be as large as 3.7 mm longitudinally in a phantom. The same group [27] also studied fiducial marker localization using a Varian Trilogy linear accelerator and found mean differences of 1.5 mm in the longitudinal direction between CBCT and kV imaging in a phantom. Since these two results are obtained from phantom studies, the localization differences cannot be attributed to patient or prostate motion. In patients, they found that 88.5%, 85.4% and 100% of localizations agreed within 3 mm in the vertical, longitudinal and lateral directions when comparing kV imaging and CBCT. However, the kV images were obtained approximately 10 minutes after the CBCT, during which, the prostate may have exhibited significant intrafraction motion [28]. Moseley et al. [20] found that 95.5%, 91.3% and 99.7% of MV fiducial localizations agreed within 3 mm with kV CBCT. In our study, the fraction of localization differences that are less than 3 mm in the vertical, longitudinal and lateral directions are 95.8%, 94.3% and 97% for the Calypso-OBI comparison and 86.1%, 84.5% and 96% for the Calypso-CBCT comparison. These results agree well with the patient data from Owens et al. The largest and most frequent disagreement between Calypso and radiographic localization is in the longitudinal direction, and the agreement is worse for CBCT than for planar kV imaging, in agreement with the results of both Owens and Moseley. The CBCT reconstruction slice thickness in the present study is 1.0 mm, less than that of Owens (2.5 mm) or Moseley (2.0 mm), and may explain why our mean differences are smaller than those observed in the earlier studies. Better resolution in the longitudinal direction would increase the accuracy of CBCT localization along that axis. Another potential source of error in CBCT localization is the imaging artifact caused by the fiducial markers in the prostate. In contrast to planar kV imaging, the blurring and streaking in CBCT images makes it more difficult to determine what constitutes a good alignment with the markers in the reference CT.

Langen et al. [19] compared marker, anatomical and contour based registration techniques using MV CT and found marker based registration to be more accurate than the other techniques. They also state “Because our patient alignments are based on fiducial marker information, they do not differ from those that would have been made if the fiducial markers were detected on an orthogonal portal image pair.” This and other studies suggest, however, that for prostate localization, the three-dimensional information may complicate the localization process and introduce more uncertainty than two dimensional orthogonal imaging. When localizing patients using 2D images, therapists are adjusting the match in only two dimensions at a time and must switch to another view to match the orthogonal image set. The only image that can be used to adjust the lateral position of the patient is the AP and likewise, only the lateral image can be used to adjust the vertical (AP) position. A lateral adjustment of the AP image does not affect the therapist’s match of the lateral images. However, when matching two 3D image sets, an adjustment in the lateral direction immediately alters the match in the coronal *and* axial registrations, leading the therapist to select a localization that may be a compromise between perfect matches in each of the two views.

One recent paper comparing planar kV imaging and CBCT for the prostate found that the precision and setup accuracy of implanted fiducial markers on 2D- 2D and 3D images was approximately the same magnitude [29]. Logadottir’s margin calculations from the setup errors yielded slightly larger values for the kV imaging than for the CBCT. A possible explanation for their result is that the kV imaging was always performed after the CBCT was acquired, increasing the likelihood that the prostate would be further from its localized position. The data presented in their study supports this conclusion, as the average shift in marker position increased with an increase in time between imaging procedures. Logadottir et al. acknowledge that it is not possible to separate differences in accuracy from differences caused by prostate motion, which is also a limitation of the present analysis.

Despite highly accurate localization techniques, margin reduction is limited by intrafraction motion [13]. In contrast to other localization technologies, Calypso has the ability to continuously track the prostate during radiation therapy. As an example, Calypso detected a 7.5 mm longitudinal shift that occurred after the localization CBCT had been obtained. This shift was verified with a subsequent CBCT and corrected. A separate group of patients treated on our SBRT prostate protocol receive a CBCT prior to and in the middle of treatment. Based on matching of implanted gold seeds, the intrafraction CBCT routinely detects shifts of 3 – 5 mm, underscoring the inadequacy of a snapshot localization at the beginning of treatment. Noel et al. [30] concluded that intermittent imaging is not sufficient for accurately predicting intrafraction motion between the two images.

## Conclusions

In patient localizations with both CBCT/kV imaging and the Calypso system, the average localization differences are less than 0.8 mm, indicating excellent agreement. Each of the three localization methods is accurate and suitable for high-precision prostate localization.

The additional volumetric information provided by CBCT is generally believed to be beneficial to patient localization. For example, in addition to localizing the prostate, CBCT provides information about the shape and location of organs at risk such as the bladder and rectum that other modalities do not. From a CBCT, therapists and physicians can quickly determine whether or not the patient has followed the pre-treatment bladder preparation instructions. For patients treated with a rectal balloon in place, a CBCT is necessary to check the volume and positioning of the balloon. We have demonstrated that there is more uncertainty in localizing the prostate with conebeam CT than with orthogonal kV images, though it is our opinion that the small increase in uncertainty in the prostate position with CBCT is not clinically significant and does not outweigh the usefulness of the extra anatomical information provided by volumetric imaging.

Calypso is unique in that it is the only localization method for prostate patients that does not require matching an acquired image with a reference, potentially resulting in more accurate localization without additional dose to the patient. However, it provides no information about the target itself or organs at risk.

## Competing interest

Timothy Solberg teaches radiosurgery courses sponsored by BrainLAB AG and receives research funding from Elekta, Ltd., Varian Medical Systems, the NIH (1S10RR028011 and R01CA139043), the DoD (PC100931) and the Cancer Prevention Initiative of Texas. Ryan Foster receives research funding from Elekta, Ltd, the NIH (R01 NS049517), and the Cancer Prevention Initiative of Texas. All other authors report no conflicts of interest.

## Authors’ contributions

RF analyzed the data and wrote the manuscript. DP treated the vast majority of the patients referenced in the study and participated in the coordination of the study and helped draft the manuscript. TS participated in the design and coordination of the study and helped draft the manuscript.

## Presented in part at

World Congress on Medical Physics and Biomedical Engineering, Munich Germany, 2009.
